# Microscopy of the Dental Tissues. New Series

**Published:** 1869-03

**Authors:** S. P. Cutler


### ARTICLE II.

### *Microscopy of the Dental Tissues. New Series.*

By S. P. Cutler M. D., A. E. G., D. D. S.,

*Continued.*

Physiology and Histology—Life may be regarded as a forced state, controlled by higher chemical endowments of matter acting in antagonism to the more normal or inorganic state of matter, hence, life is only a temporary condition of inorganic matter, brought up from the inorganic and sustained for a time, and then returned again to its original condition whenever any disturbance breaks up or greatly disturbs organic laws or forces. One pole or axis of organisms may be infered to rest in the organic, and the other in the inorganic kingdom, more especially in animal organisms and in constant antagonisms, that of organic being only a special condition, and the inorganic general and universal condition of matter. The one only temporary, the other eternal, the one is universal the other local and special, the one always predominant, the other only temporarily so, hence all life must sooner or later end in death or a return to the more normal condition. Physiology sustains life, and pathology drags down towards death or decay, vis inertia or equilibrie.

The plant rears organisms from the inorganic kingdom, from the binarys, as water, carbonic acid, and ammonia, and others of less moment, such as the minerals proper, sulphur, iron, phosphorus, lime, potash, soda, and some others.

The plant may decay and give back its elements to their original condition direct, or pass them back through the animal creation to their former state.

After an animal organism is formed, it must be sustained from other organisms, as it cannot be sustained to any extent from the inorganic directly, that is organic food enters the animal organism, and leaves in the original binary condition, in other words, these elements are returned to their binary inorganic condition before leaving the body. This change, or process takes place in the cells and fluids of the body every where. This change from organic to inorganic taking place constantly in the tissues not only nourishes the tissues, but at the same time developes vital fire and magnetism in the life forces, without which no vital phenomena or vis vite could be developed or sustained at all.

I have given some of the chemical and vital changes that take place in the cell structure proper, it will now be necessary to give the changes that take place in cell contents. These belong to the ternary group, as oils, starch and sugar, all of which are combustible in different degrees. Even sugar contains 12 atoms of carbon and 11 atoms of oxygen and hydrogen each, or 11 atoms of water. Now, there is only 11 atoms of oxygen in sugar, this just neutralizes the amount of hydrogen, leaving the 12 atoms of carbon to be oxidized or burned in the organism to develop vital fire. This carbon will unite with just twice its amount by atom of oxygen, that is, 24 atoms of oxygen will unite with 12 atoms of carbon to form carbonic acid, which sets free a large amount of heat. Sugar burns with some flame which leads to the conclusion that the oxygen contained may be in the form of water chiefly, and that sugar is a hydrate of carbon, or a carboret of water.

Starch containing one atom less of water than sugar, two atoms more of oxygen, combines with starch in combustion, and in consequence developes one twelfth part more heat than sugar combustion. Now, these substances burn with flame, some of the atoms of hydrogen are not in the form of water, and some of the carbon is in the form of carbonic oxide, which has to take only one atom more of oxygen to become carbonic acid. In this case carbonic acid and water are chemically formed, such is the case either way, as the elements of water as such are set free as water when the carbon is consumed by oxidation.

Oils and fats are more combustible and burn with more flame and greater heat, than either of the former from the large amount of the combustibles to the supporters of combustion contained. Margaric acid contains carbon 34, hydrogen 32, and oxygen 4, combined with glycerine, the base of the fat, carbon 6, oxygen 6, and hydrogen 8, add this to the margaric acid and we have carbon 40 atoms, hydrogen 40 atoms, and oxygen only 10 atoms. How these 10 atoms of oxygen are combined in margaren is not definitely known. As a matter of convenience I will suppose there to be 10 atoms of water, which leaves thirty atoms of hydrogen and 40 atoms of carbon to be oxidized, the 30 atoms of hydrogen combines with 30 atoms of oxygen when in a state of incondescence burns with a pale flame, and gives out a large amount of heat as the two gases are reduced to a liquid, hence a great condensation and giving up of latent, to become sensible and free heat. The 40 atoms of carbon combines with 80 atoms of oxygen to form carbonic acid gas, one volume of carbon vapor and one volume of oxygen, condensed to one volume of carbonic acid.

It is the condensation of oxygen that develops all the heat, as the carbon vapor has to be so converted by heat taken from the oxygen in the first process of sensiblecombustion, and insensible combustion is precisely the same thing, only, it is a slower process and the developed heat does not amount to incondescence of the combustible solid atoms of carbon and hydrogen. Stearine or stearate of glycerin contains the number of atoms of carbon and oxygen, and two more of hydrogen, hence it generates more heat than margarin. Oleine or oil, oleate of glycerine, contains the same number of atoms of oxygen as either of the others, and two more each of carbon and hydrogen than the stearine, which, renders it a greater heat generator than either of the others, one atom of oleine combining with 116 atoms of oxygen. Thirty two atoms of uncombined hydrogen, and 42 atoms of carbon combine with the above named quantity of oxygen atoms, atom for atom of hydrogen, and one atom of carbon, to 2 atoms of oxygen forming water and carbonic acid. Carbonic acid contains its own volume of oxygen and a specific gravity about one and a half times that of oxygen, hence great heat is set free in the formation of carbonic acid. Oxygen and hydrogen condense to a liquid and a much greater amount of latent heat set free by the hydrogen combination than the carbon. The difference in the heat generated in the combustion of oil in the oxidation of carbon alone, is three times greater than that of starch or sugar, the,combustion of the 32 atoms of hydrogen added makes the calorific difference very great, the exact difference not being easily ascertained by deductions alone, but only by actual experiments. It will readily be seen that the fats and oils constitute the chief calorifacients of animal organisms, calidum, animate or inanimate. It will be seen that the cell contents, as a general thing suffer oxidation to a greater extent than cell structures themselves, which are constituted of the compound radical type, proteine, an ozotized element.

Formula—(C 36–H 25–N 4–O 10– 2HO.) Mulder which which differs but slightly from Leibig's formula, proteine may be rgarded as the radical of all the nitrogenised histological elements of the animal organism, such as albumen, fibrin and cosein that enter into nutrition.

In the destructive oxidation of the cells there is only 3 atoms of hydrogen to suffer oxidation, supposing the 4 atoms of nitrogen to be united with 12 atoms of hydrogen to form ammonia, and the other 10 atoms to be neutralized by the 10 atoms of oxygen, that leaves the 36 atoms of carbon to be burned by oxidation. There is then 36 atoms of carbon and 3 atoms of hydrogen to suffer oxidation in the cell structures so that when an atom of the elements of cell structure is burned, more than three times the amount of latent heat is set free than when an atom of starch or sugar is burned, either in or out of the body, though it falls far short of the oil group, even if oxidised in the same ratio, which however cannot be the case, as there is much more vital resistance to be overcome in the oxidation of cells than in their contents or in the circulating fluids. Whether or not the proteine compounds contains ammonia, as such, in the structures of the body, cannot be easily determined, though ammonia enters into their combinations, and is also formed by the breaking up of such structures, or whether all the oxygen is in the form of water and carbonic acid, as such, is not known, though they both enter into their combinations, and both leave as such in their destruction. No matter how the oxygen is combined a given amount has to be absorbed before any breaking up of tissues can take place. If any free sulphur or phosphorus are found to exist in the body, they will undergo oxidation and will be an additional source of animal heat; if they enter the organism in combination, no heat is derived from them, or any other mineral element so entering. Again, nothing gives up any caloric to the organism but oxygen, free oxygen, taken in by respiration, not in combination, as taken in in the form of food,

There is no other source of heat, except solar or artificial, absorbed from without, or taken in in the form of warm food or drink. In hot climates more solar heat is absorbed than in cold climates, and it is but reasonable to suppose that much more food of combustion is needed in cold than hot climates. The same may be said of clothing and exercise.

*To be Continued.*

---

